# Completeness of regional cancer registry data in Northwest Russia 2008-2017

**DOI:** 10.1186/s12885-023-11492-z

**Published:** 2023-10-18

**Authors:** Anton Barchuk, Rustam Tursun-zade, Ekaterina Nazarova, Yuri Komarov, Ekaterina Tyurina, Yulia Tumanova, Alexey Belyaev, Ariana Znaor

**Affiliations:** 1 Institute for Interdisciplinary Health Research, European University at St. Petersburg, Shpalernaya Ulitsa 1, 191187 St. Petersburg, Russia; 2 NN Petrov National Medical Research Center of Oncology, Pesochny, Leningradskaya Ulitsa 68, 197758 St. Petersburg, Russia; 3 ITMO University, Kronverkskiy Prospekt, 49, 197101 St. Petersburg, Russia; 4 OPIK, Departamento de Sociologia y Trabajo Social, Universidad del País Vasco (UPV/EHU)), Barrio Sarriena s/n, 4894, 69007 Leioa, Spain; 5 Cancer Surveillance Branch, International Agency for Research on Cancer, 25 avenue Tony Garnier, 69007 Lyon, France

**Keywords:** Cancer registry, Data quality, Completeness, Timeliness, Northwest of Russia

## Abstract

**Background:**

A national framework for population-based cancer registration was established in Russia in the late 1990s. Data comparability and validity analyses found substantial differences across ten population-based cancer registries (PBCRs)in Northwest Russia, and only four out of ten met international standards. This study aimed to assess the completeness of the PBCR data of those registries.

**Methods:**

Qualitative and quantitative methods recommended for completeness and timeliness assessment were applied to the data from ten Russian regional PBCRs in Northwest Russia, covering a population of 13 million. We used historic data methods (using several European PBCRs reference rates), mortality-to-incidence ratios (M:I) comparison, and death certificate methods to calculate the proportion of unregistered cases (Lincoln-Petersen estimator and Ajiki formula).

**Results:**

Incidence rate trends of different cancer types were stable over time (except one region - Leningrad oblast). A slight drop in incidence rates in older age groups for several sites in the Northwestern regions was observed compared to the reference from European countries. Comparing M:I ratios against five-year survival revealed systematic differences in Leningrad oblast and Vologda oblast. Assessment of completeness revealed low or unrealistic estimates in Leningrad oblast and completeness below 90% in St. Petersburg. In other regions, the completeness was above 90%. The national annual report between 2008-2017 did not include about 10% of the cases collected later in the registry database of St. Petersburg. This difference was below 3% for Arkhangelsk oblast, Murmansk oblast, Novgorod oblast, Vologda oblast and the Republic of Karelia.

**Conclusions:**

Eight out of ten regional PBCRs in Northwest Russia collected data with an acceptable degree of completeness. Mostly populated St. Petersburg and Leningrad oblast did not reach such completeness. PBCR data from several regions in Northwest Russia are suitable for epidemiological research and monitoring cancer control activities.

**Supplementary Information:**

The online version contains supplementary material available at 10.1186/s12885-023-11492-z.

## Background

Population-based cancer registration is a unique and reliable source of structured information for cancer surveillance, and research [1]. The framework and instructions for Russia’s regional population-based cancer registries (PBCRs) were developed in the late 1990s [2]. The national cancer statistics report is published annually and has been available online since 2007. However, to ensure accurate and reliable cancer statistics, the quality of initial PBCR data needs to fulfil specific criteria. Researchers apply four key quality dimensions to evaluate the PBCRs data: comparability, validity, completeness, and timeliness [3, 4].

Our previous study focused on the comparability and validity of the data from ten Russian regional PBCRs covering a population of approximately 13 million [5]. It showed that the overall level of comparability and validity in four out of ten PBCRs in Northwest Russia met international standards. However, the differences in the data comparability and validity between the regions were substantial. Moreover, the analysis of completeness and timeliness should supplement the comprehensive assessment of PBRCs’ data quality. Although all four quality indicators are essential, completeness has outstanding methodological diversity and complexity [4]. Both quantitative and qualitative methods are used to assess the overall completeness. Information from different sources is also crucial for the quantitative assessment of completeness. Reports from several high-quality PBCRs in Europe and Israel revealed completeness above 90% [6–11].

The presence and the number of independent sources for PBCR are often limited to different settings and healthcare systems. In countries with developed modern healthcare systems, PBCRs collect information through different sources. For example, In Norway, data are provided by hospitals, pathological laboratories, general practitioners, and the National Statistics Office (death certificates) [6]. In Finland, PBCR receives notifications from multiple sources: hospitals, physicians, pathology and haematology laboratories, the Population Register Centre, and the National Statistics Office [7]. In Bulgaria, PBCR receives information from notifications of new cancer cases, hospital discharge records, pathology reports, other diagnostic laboratory results, and death certificates [10]. In Ukraine, not all regions had access to cause of death information [12], and some quantitative methods for completeness could not be applied. In Hungary, the death certificate information is available from the National Statistics Office for 85-90% of cases. However, cases unique to death certificate databases are not recorded in the PBCR database [13], making some quantitative methods for completeness assessment not feasible.

The access to sources of information defines PBCR’s ability to apply different methods and assess cancer registry completeness. While several sources are virtually available in Russia [5], two sources can be reliably and explicitly identified in the PBCR database: clinical notifications and death certificates, allowing for quantitative completeness assessment. Still, it has not been performed before

Incomplete cancer registry data adds bias to cancer burden assessment. Cancer incidence and mortality trends analysis in Russia [14, 15] that guides national cancer control policies may suffer from incomplete registry data. Consequently, biased estimates can lead to suboptimal cancer control policies and resource allocation. There is also a trade-off between different quality indicators, i.e., avoiding sources of information with limited validity that require extensive checks and corrections is often tempting. Still, it would probably increase the incompleteness of the data. PBCR often needs sufficient time to collect information about the cancer cases occurring in the region from several sources. The Russian cancer registry system emphasises the production of timely reports, which can compromise data completeness [2].

This study aimed to assess the completeness of ten PBCRs in Northwest Russia.

## Methods

This report follows recommendations for completeness assessment and applies several qualitative and quantitative methods [4]. Completeness is the degree to which the PBCR covers all of the incident cases in the target population. Qualitative and semi-quantitative methods measure the degree of completeness relative to other registries and sometimes overlap with validity methods. These include the historic data methods (stability of incidence rates over time, comparison of incidence rates with similar populations, the shape of age-specific curves, and incidence rates of childhood cancers) and mortality-to-incidence ratios (M:I). Quantitative methods provide a numerical evaluation of completeness in percentages and include capture-recapture and death certificate methods.

### Data

We analysed data from the ten PBCRs databases of eleven regions of Northwest Russia (the Arkhangelsk oblast (including the Nenets Autonomous Okrug), the Murmansk oblast, the Republic of Komi, the Republic of Karelia, the Pskov oblast, the Kaliningrad oblast, the Leningrad oblast, the Novgorod oblast, the Vologda oblast, St. Petersburg) extracted in December 2019 [5].

The current state and procedures of cancer registration in Russia were in-depth described in one of our previous reports [2]. In brief, regional cancer registries established in all federal regions collect information on all malignant and in situ neoplasms (ICD-10 codes C00-96 and D00-09) from notification forms that all physicians and hospitals must provide to PBCR. PBCRs are also entitled to regularly link records with mortality records to follow up on the vital status. In practice, PBCRs are often part of a regional cancer hospital, and data collection can also (but not necessarily) involve the local hospital registry. The information should be reported by all other regional medical facilities in the region in the form of paper notifications. Information transfer may not be performed in case of migration or death in different regions.

Our previous report described regions in Northwest Russia [5], but we obtained additional information about cancer registries in the regions (Table 1). Registries were established during the 1990s except in Novgorod Oblast (2003), Vologda Oblast (2005) and Arkhangelsk Oblast (2000). Most of the registries were based in the regional cancer hospitals (Oncology Dispensaries), except Saint-Petersburg and Kaliningrad Oblast, where registries are part of the regional medical statistics service. All the regions somehow established the mortality linkage with mortality data. However, it was manual in half of them. In other words, cancer registry personnel or regional oncologists manually reviewed death certificate records at civil registries to detect 1) cancer patients from the registry who have died and 2) death certificates that mention cancer. The semi-automatic procedure involves searches in electronic databases of the state insurance system (Obligatory Medical Insurance) and/or civil registries. Active traceback of notifications based on death certificate only (DCN) was reported in 6 out of 10 PBCRs.

**Table 1:** Description of the ten population-based cancer registries in Northwest Russia (registry survey data)

Region	PBCR foundation year	Current institution	Database backup	Linkage mortality data	Active traceback of DCN cases
Arkhangelsk Oblast	2000	Arkhangelsk Oblast Oncology Dispensary	Daily	Semi-automatic, monthly	Yes
Kaliningrad Oblast	1991	Kaliningrad Oblast Oncology Center	Daily	Manual and semi-automatic, monthly	No
Republic of Karelia	1996	Republic of Karelia Oncology Dispensary	Daily	1996-2015 - manual, monthly; since 2016 - semi-automatic, weekly	Yes, 2-3 weeks after the death
Republic of Komi	1996	Republic of Komi Oncology Dispensary	Monthly	Manual, monthly	Yes, 2-3 months after the death
Leningrad Oblast	1998	Leningrad Oblast Oncology Dispensary	Weekly	Manual, monthly	No
Murmansk Oblast	1993	Murmansk Oblast Oncology Dispensary	Daily	Semi-automatic, monthly	Yes
Saint-Petersburg	1993	Medical Information and Analytical Center	Daily	Semi-automatic, monthly	No
Novgorod Oblast	2003	Novgorod Oblast Oncology Dispensary	Every 3-months	Semi-automatic, monthly	Yes
Pskov Oblast	1995	Pskov Oblast Oncology Dispensary	Monthly	Manual, monthly	Yes
Vologda Oblast	2005	Vologda Oblast Oncology Dispensary	Daily	Manual, monthly	No

PBCR - population-based cancer registry; DCN - case, notifies with a death certificate; dispensary - regional cancer hospital

PBCRs data were subject to the multistep conversion and cleaning procedure using “IARC/IACR Tools for Cancer Registries” software [16]. We applied international rules for multiple primary cancers (ICD-O) to eliminate duplicates and assigned the corresponding ICD-10 codes to all cases. About 1.27 million cancer cases for the whole period and 0.57 million for the most recent period, 2008 - 2017, were used in the analysis. All ages and ICD-10 diagnoses C00-96 were included in the analysis. In the graphs, we used data from 1993, and in the main analysis, the most recent periods, 2008 - 2017. We extracted mid-year population estimates and cancer mortality rates by cause, sex, 5-year age group, and region from the Russian Fertility and Mortality Database (RFMD). RFMD is identical to the national statistics data collected by the Russian Federal State Statistics Service (RSSS) [17]. We calculated age-standardized rates (ASRs) per 100,000 per calendar year using the Segi-Doll 1960 world standard population [18]. We used these datasets previously to assess data comparability and validity [5].

### Historic data methods

We used cases registered between 1993 and 2017 to assess the stability of incidence rates over time. To perform a preliminary assessment, we produced the plots for the number of cases (C00-C96) and age-standardized incidence rates (ASRs) per 100,000 per calendar year and ASRs per 100,000 for haematological malignancies. Then, we compared cancer incidence rates for age groups 0-4, 5-9 and 10-14 with the corresponding reference intervals based on deciles for childhood cancer published in IARC “Cancer Incidence in Five Continents (CI5)” volume XI [19]. Finally, we examined the shape of age-specific curves (2008-2017)) on the arithmetic and semi-log scale against the curves from several European cancer registries from “Cancer Incidence in Five Continents” volume XI (2008-2012): (Bulgaria, Czech Republic, Latvia, Lithuania, Estonia and Poland (Cracow, Lower Silesia, Kielce, Podkarpackie).

### Histological verification of diagnosis and M:I ratios

Our previous report summarised some quality indicators: the proportion of morphologically verified cases (MV%), the proportion of cases registered with information available from death certificates only (DCO%), and the mortality-to-incidence ratios (M:I) [5]. For the current report, first, we plotted M:I ratios (2008-2012 and 2013-2017) against similar estimates from several registries in Eastern Europe (Bulgaria, Czech Republic, Poland, Latvia, Lithuania, Estonia, data from Globocan [20] and “Cancer Incidence in Five Continents (CI5)” volume XI [19]) and Norway (data from Nordcan [21, 22]) in 2008-2012. We plotted M:I ratios from 2013 to 2017 against one minus 5-year survival (calculated using the Ederer II method [23, 24]) for cases diagnosed from 2008 to 2012. Finally, we used an arbitrary minimal value of 10% to define a relevant absolute difference between survival and M:I [25].

### Quantitative methods

We applied the death certificate method to calculate completeness as the proportion of cases registered and the sum of registered and unregistered cases.

Two sources are available in PBCR databases - “official cancer case notification” and “death certificate notification”. Therefore, we used the following assumptions to distinguish between the two: 1) if the information on the date and cause of death was available, we considered the case as having information from the death certificate, 2) if the date of diagnosis was not equal to the date of death, we considered the case as having clinical notification, 3) if the date of diagnosis was equal to the date of death, and additional clinical information such as stage or treatment was present, we considered the case as having clinical notification. Additionally, we distinguished DCI cases. We assumed that “the proportion of cases for which the information was received first via a death certificate notification” (DCN) approximates “the proportion of cases for which the first information comes via a death certificate, and, without it, the case would not have been identified” (DCI). So all death certificate only (DCO) cases and cases labelled as “initially notified through death certificate” were considered DCI.

To obtain the quantitative completeness estimates, we applied the method based on death certificate cases [4]. In the situation with two sources, it was equivalent to the Lincoln-Petersen estimator and could also be obtained from capture-recapture logit models [26].

To obtain a numerical estimate of undetected cases (UC), we assumed that the proportion of unregistered cases that die — $\frac{DCI}{DCI + UC}$, is the same as the proportion of registered cases that die — $\frac{RD}{RD+RA}$, where RD — registered cases that die, RA — registered cases that are alive. Thus, $UC=\frac{DCI\times {RA}}{RD}$ and the degree of completeness is

$$\begin{aligned} \frac{RA+RD+DCI}{RA+RD+DCI+UC}. \end{aligned}$$

Alternatively, we applied the Ajiki formula [27] to estimate completeness. We used mortality-to-incidence ratios with an independent source for the number of deaths (based on civil registry statistics). As a result, we calculated the completeness proportion using the following formula:

$$\begin{aligned} \frac{1-DCI\times {\frac{1}{M:I}}}{1-DCI.} \end{aligned}$$

### Timeliness

Timeliness is another data quality dimension linked to completeness. After the initial annual report is published, properly functioning cancer registries update the databases [6]. Russia publishes the national cancer report that includes regional data in the late autumn of the following year, but aggregated data are being collected in January [2]. Aggregated data is collected using special forms that include the number of cases by age (18 age groups) and gender for the ICD-10 cancer diagnoses and some additional information. It is considered too early for most cancer registries that aim at providing annual reports in one to three years [3]. To assess the effect of early reporting, we compared the absolute number of cases from Northwestern Russian regions in 2008-2017 available in the ten annual national reports to the number of cases in our database extracted in December 2019.

## Results

**Fig. 1:**
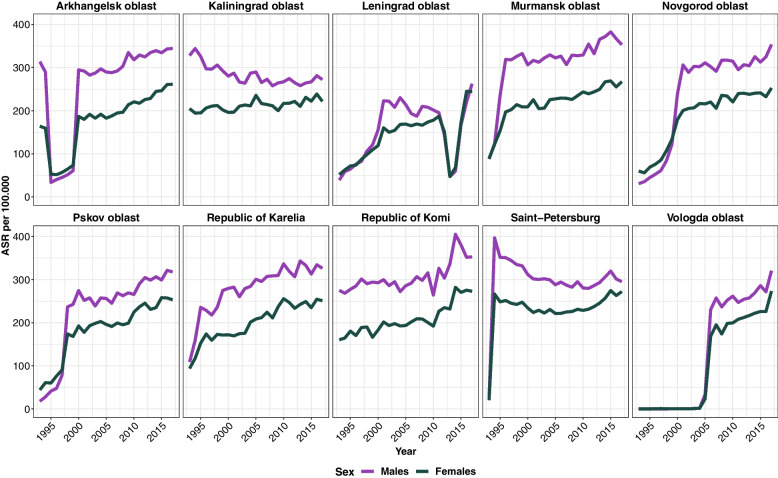
Age-standardized cancer incidence rates per 100,000 for regions in Northwest Russia (all cases with behaviour code = 3, including non-melanoma skin cancer, world population Segi-Doll, 1960)

Full-scale cancer registration started in 1991 in the Republic of Komi and Kaliningrad oblast, in 1993 in St. Petersburg, in the late 1990s early 2000s in other regions except for Vologda oblast (Fig. 1 and Supplementary Fig. S1). In Vologda oblast, registration of solid cancer types started in 2005, and for haematological malignancies only in 2013 (Supplementary Fig. S2). In addition to overall low incidence rates in Leningrad oblast, a drop in the incidence rates was evident in 2014-2016.

Childhood cancer rates were generally within the reference bounds, with a slight deviation from the reference observed in some regions (Supplementary Table S1). Specifically, in both periods, childhood cancer rates at ages 10-14 years were higher for boys and girls in Novgorod oblast and girls in Pskov oblast. Rates were also above the reference range for boys aged 5-9 in Novgorod oblast and below the range in Leningrad oblast.

ASRs trends of different cancer types were stable over time, as seen on logarithmic scales (Supplementary Fig. S3). Overall, the shapes of age-specific curves were similar to those of the other European countries (Supplementary Fig. S4). A slight drop in incidence rates in older age groups for several sites in the Northwestern regions was observed compared to European countries.

**Fig. 2:**
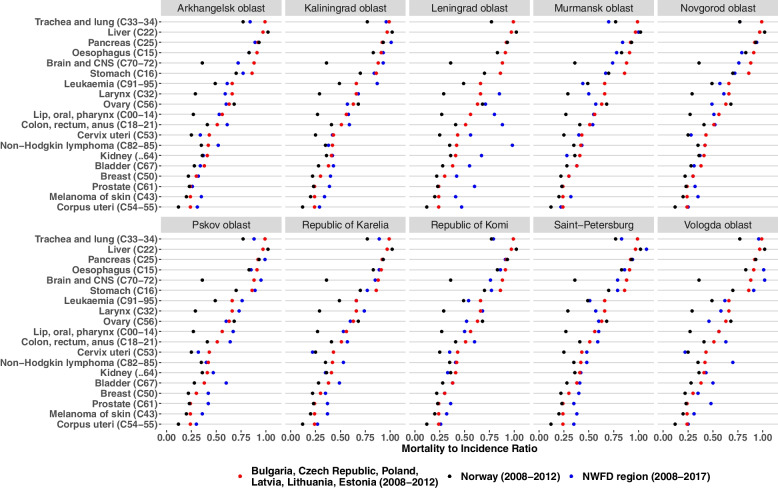
Comparison of mortality-to-incidence ratios by cancer site, regions of Northwest Russia in 2008-2017, Eastern European Countries (data from Globocan [20]) and Norway (data from Nordcan [21]) in 2008-2012

M:I ratios for most cancer types were higher in the Northwestern Russian regions than in other countries in 2008-2012 (Fig. 2). The difference was clear for the brain and CNS, bone and cartilage cancers. M:I ratios for breast and colorectal cancers were also higher in Northwest Russia. However, M:I ratios for ovarian cancer were slightly lower across most of the Northwestern regions and for kidney and lung in some of the regions (Murmansk oblast, Novgorod oblast). M:I ratios were systematically higher across all cancer types and periods in Leningrad oblast.

**Fig. 3:**
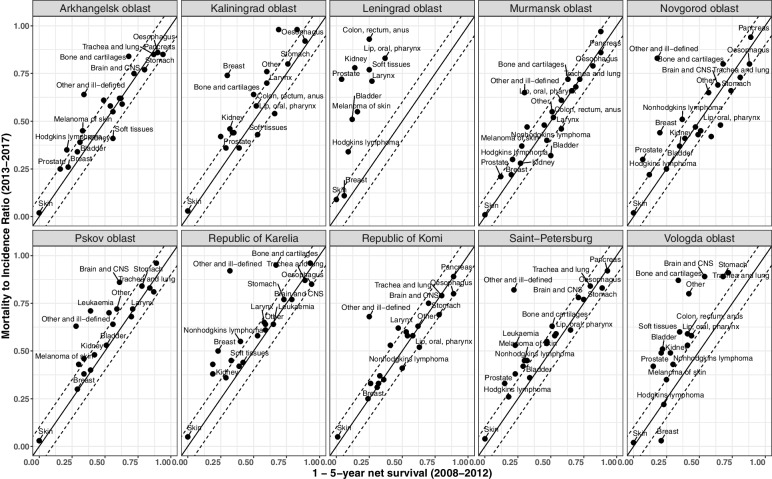
Mortality-to-incidence ratios (2013-2017) versus one minus five-year relative survival (based on diagnoses in 2008-2012) in men, regions of Northwest Russia

**Fig. 4:**
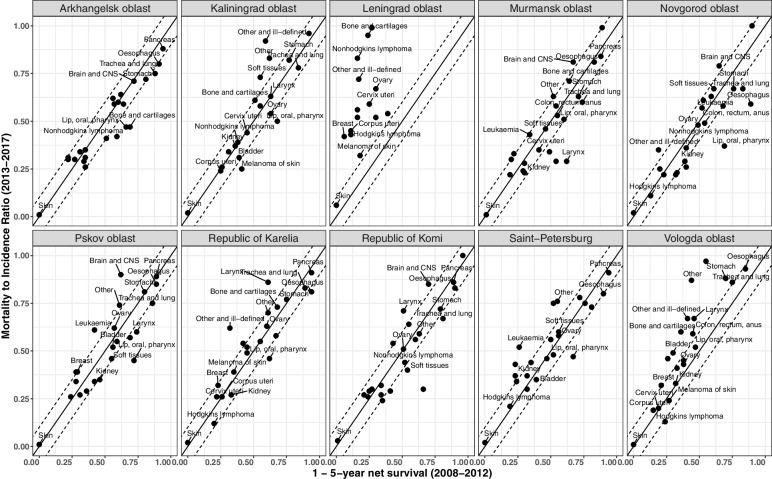
Mortality-to-incidence ratios (2013-2017) versus one minus five-year relative survival (based on diagnoses in 2008-2012) in women, regions of Northwest Russia

The comparison of M:I ratios from 2013 to 2017 against one minus five-year survival of cases diagnosed from 2008 to 2012 (Fig. 3 for men and Fig. 4 for women) revealed systematic deviance from the reference line Y = X at least in two regions. In Leningrad oblast, survival proportions were much higher than expected by M:I for most cancer types in men and women. We observed similar differences for several cancer types in Vologda oblast. There were outliers in all the regions (e.g. breast cancer in Kaliningrad oblast), but in most cancer types, the difference did not exceed the prespecified limit of 0.1.

Assessment of completeness revealed low or unrealistic estimates in Leningrad oblast and a lack of completeness in St. Petersburg (Table 2). Completeness in the other regions was above 90% from 2008 to 2017. Completeness for all cancer sites in eight Northwestern regions (excluding St. Petersburg and Leningrad oblast) was above 90%. Lincoln-Petersen estimator was below 90% only for skin cancer - 86.8%, corpus uteri - 88.5%, Hodgkin lymphoma - 79.1%, leukaemia - 89.4% and other and ill-defined cancers - 89.5% (Supplementary Table S2). In St. Petersburg, completeness estimates were below 90% for most cancer types (Supplementary Table S3). In Leningrad oblast, completeness evaluation was inconsistent, with completeness below 60% for some cancer types (Supplementary Table S4).

**Table 2:** Data sources and completeness estimates in the regions of Northwest Russia, 2008-2017, all sites except non-melanoma skin (C00-C96 without C44)

Region	Cases	Deaths*			Data sources (%)				Completeness (%)	
		Period	Cohort	M:I ratio†	C/P	C/P and D‡	DCO	DCI	Lincoln-Petersen estimator	Ajiki formula
Arkhangelsk oblast	46,519	26,831	25,537	0.58	44.2	47.8	4.5	8.0	93.3	93.6
Kaliningrad oblast	30,964	19,231	16,967	0.62	45.1	50.7	0.9	4.3	96.5	97.3
Leningrad oblast	44,095	39,825	15,934	0.90	63.7	26.6	6.5	9.8	72.9	98.8
Murmansk oblast	28,459	13,898	12,584	0.49	54.9	40.3	2.1	4.8	92.9	94.7
Novgorod oblast	25,945	13,822	11,946	0.53	53.6	39.6	6.2	6.7	90.4	93.7
Pskov oblast	25,651	16,522	14,290	0.64	44.2	50.2	3.6	5.6	95.6	96.7
Republic of Karelia	25,548	15,083	13,734	0.59	45.9	46.0	2.6	8.1	92.6	93.9
Republic of Komi	30,924	17,078	15,868	0.55	48.5	42.1	4.6	9.4	89.6	91.5
Saint-Petersburg	219,593	129,045	118,419	0.59	44.8	36.1	17.6	19.1	79.4	83.5
Vologda oblast	38,955	25,693	20,498	0.66	47.2	44.1	8.4	8.7	91.2	95.1

C/P - clinical/pathological notification only; C/P and D - clinical/pathological notification and death certificate; DCO - cases registered based on death certificates only; DCI - cases initially registered based on information from the death certificate and further investigated.

* - period cancer deaths were obtained from the civil registry for 2008-2017; cohort cancer deaths were obtained from the cancer registry database for patients diagnosed in 2008-2017.

†- Mortality to incidence ratio was based on the number of deaths from the civil registry.

‡- all DCI cases were excluded, including those with clinical/pathological information

The comparison of the annual report and registry database between 2008-2017 showed that about 10% of cases (or 23,304 cases) were not included in the initial national report for St. Petersburg. On the other hand, there were 19% fewer cases in the Leningrad oblast registry (Table 3). The differences for Arkhangelsk oblast, Murmansk oblast, Novgorod oblast, Republic of Karelia, and Vologda oblast were below 3%. Early reporting similarly affected most cancer types in the other eight regions, except liver and haematological malignancies (initial number overestimated) (Supplementary Table S5). The number of cases for most cancer types was underestimated in the national report for St. Petersburg, and (Supplementary Table S6), on the other hand, was lower in the Leningrad oblast registry database than in the national report. (Supplementary Table S7).

**Table 3:** Cancer cases from the registry database and the national annual report in the regions of Northwest Russia, 2008-2017

	Number of cases		Difference	
Region	Registry	National report	Absolute	Relative (%)
Arkhangelsk oblast	51,610	50,953	657	1.3
Kaliningrad oblast	35,611	34,074	1,537	4.3
Leningrad oblast	48,535	57,556	-9,021	-18.6
Murmansk oblast	30,839	30,458	381	1.2
Novgorod oblast	29,296	29,171	125	0.4
Pskov oblast	30,144	29,169	975	3.2
Republic of Karelia	27,863	27,104	759	2.7
Republic of Komi	33,216	31,694	1,522	4.6
Saint-Petersburg	237,810	214,506	23,304	9.8
Vologda oblast	44,495	43,435	1,060	2.4

## Discussion

Our study is the first comprehensive assessment of completeness in ten PBCRs of Northwestern regions of Russia. This study complements the assessment of comparability and validity published earlier [5] and provides the basis for using PBCRs datasets in epidemiological studies. Completeness is an essential criterion for PBCRs data quality, and it was above 90% in most regions (except St. Petersburg and Leningrad oblast). Relatively low completeness in St. Petersburg and Leningrad oblast is likely due to the size of the population and direct access to multiple cancer diagnostics and care facilities that do not notify PBCRs. Another important finding is the 10% difference in the number of cases in the PBCR database and the national report for St. Petersburg compared to other regions.

A slight drop in incidence rates in older age groups for several sites in the Northwestern regions compared to European registries could indicate a lack of completeness in older ages. The lack of completeness in older age groups was described in other countries, explained by the limited invasive diagnostics and non-referral, e.g. for patients with suspected breast cancer ([28, 29]). Given the overall lower life expectancy in Russian regions and the burden of other chronic diseases, that could explain the drop in age-specific curves.

In Northwest Russia, M:I ratios for less lethal cancer types (e.g., breast and colorectal cancer) were generally higher than in Eastern European countries. We expected higher M:I ratios if the incidence data is incomplete (as with the Leningrad oblast case). Still, incidence depends on diagnostics and screening practices, affecting survival [30]. M:I ratio for more lethal cancer types (e.g., lung and oesophagus) in several Northern regions (e.g., Arkhangelsk oblast and Murmansk oblast) were more similar to M:I ratios in Norway (0.77) than to M:I ratios in Eastern Europe. The high annual volume of specific cancer diagnostics procedures (x-ray, computer tomography, and endoscopy) in Murmansk oblast and Arkhangelsk oblast could be one of the explanations for this difference in M:I (personal communication, Aug 2022). Overall, M:I ratios in Northwest Russia were similar for most cancer types compared to Eastern Europe, suggesting similar survival patterns.

Difference between the M:I ratios and the survival did not exceed the predefined limit, indicating reasonable completeness for most cancer types in all the regions except Leningrad oblast and Vologda oblast. Leningrad oblast PBCR database was incomplete during 2014-2016, which was evident in the visual assessment of ASRs trends. Technical difficulties (software transition) and lack of resources (and staff) may have caused the Leningrad oblast’s data loss. Nevertheless, we included this database in our analyses to evaluate how different approaches to estimating completeness were performing in such a setting.

A quantitative completeness assessment showed that completeness was above the reasonable limit of 90% in eight regions out of ten. Lincoln-Petersen estimator provided a realistic estimate of completeness in the Leningrad oblast, and both methods suggested a lack of completeness in St. Petersburg. It is worth mentioning that the Lincoln-Petersen estimator provided a much more realistic estimate of completeness in the Leningrad oblast. Nevertheless, both methods provided similar results in St. Petersburg, suggesting that the assumptions for those methods probably hold. The Ajiki formula uses the M:I ratio, where mortality estimates are based on the independent source, the death registration system, while the Lincoln-Petersen estimator relies on the number of cases and deaths registered at PBCR. Some differences in estimators arise from the quality of mortality linkage in PBCR. At the same time, in case of database losses (like in Leningrad oblast), the M:I ratio becomes unrealistically high, limiting the use of this formula for completeness assessment.

Finally, despite early reporting, our timeliness assessment showed that the national annual report provided more complete statistics for smaller regions. However, it did not include about 10% of cases collected in St. Petersburg later. One month is insufficient to provide an accurate report from the previous year, and this practice should be changed. In addition to that, hematological malignancies and liver cancer estimates were overestimated in the national report. Probably the reason was the number of duplicates for hematological malignancies recorded as primary tumors and the misclassification for liver cancer when primary liver cancer cases were recorded initially for cases with other cancer types and metastases to the liver.

Our study has several limitations. We have already mentioned the lack of independent sources of cancer registrations in Russia. The cancer registration procedures in Russia do not allow separate notifications from pathology laboratories - all cases with pathological notification also have clinical notification. A similar situation was reported in Bulgaria [10]. In contrast, PBCR data completeness assessment in Norway [6] and Singapore [31] took advantage of capture-recapture models using three independent sources. Capture-recapture and related death-certificate methods depend on the assumption about source independence and probability of capture. Log-linear and logit modelling can improve our estimations, but we still depend on the available data variables, and unobserved characteristics associated with capture cannot be accounted for [4]. Another possible method to assess completeness is an independent case ascertainment, as used in Finland [7] and Iceland [32].

Another limitation of quantitative completeness assessment is the definition of the DCI proportion based on our assumptions about registry procedures. First, it is unclear if all DCI cases were accounted for in the database, and second, low DCO proportion (which is a part of DCI) may result from inefficient linking with the death certificate database. Finally, an autopsy practice (e.g. following more than 60% of the deaths in the Arkhangelsk oblast [5]) could be one of the reasons for the inflated DCO proportion.

Despite all these limitations, the analysis presented in our paper utilised most of the semi-quantitative and quantitative methods used for completeness assessment. We could not apply the flow method [4] since the registration date was unavailable in our database during our analysis. Still, as far as we know, it is available in some PBCRs and can also be used for completeness assessments.

In conclusion, more than half of the regional population-based cancer registries of Northwest Russia collect data with an acceptable degree of completeness (above 90%). The most populated regions (St. Petersburg and Leningrad oblast) do not have the same degree of completeness. Several reasons could explain the lack of completeness. First is the less centralised cancer care system in both regions, where patients can choose several federal, regional, and private hospitals providing cancer care. Second, software transition resulted in database loss, and a lack of staff in the registry limited the up-to-date data collection in Leningrad Oblast. Third, St. Petersburg’s population requires more cancer registry personnel to process all the cases.

Several important recommendations should be drawn from this report. Additional sources for PBCR can be explored, for example, pathology laboratories and hospital registries. Automatic linkage with mortality data sources could also improve data quality, but it requires access for PBCR personnel to these data sources. Finally, the national report would benefit from the additional time given to PBCR to collect the data.

Considering other quality indicators (comparability and accuracy), cancer registry data from several PBCRs in Northwest Russia is sufficient for epidemiological research and monitoring cancer control programs. Our project has already improved some aspects of PBCR data collection in the Northwestern region. However, despite overall good completeness and validity, PBCRs have challenges in comparability due to non-standard practices and classifications. Therefore, further implementation of ICD-O-3 is one of the main goals for the near future. We also hope other Russian regions will conduct quality assessments and use data to assess cancer incidence and survival using individual-level data.

### Supplementary Information

**Additional file 1.** Supplementary Material. Supplementary material with the additional figures and tables.

## Data Availability

All analyses were conducted in R, and aggregated study data is available upon request (contact Anton Barchuk at the European University at St. Petersburg or Yuri Komarov NN Petrov National Medical Research Center of Oncology).
