# Supplementary material for: Completeness of regional cancer registry data in Northwest Russia 2008-2017
Source: BMC Cancer. 2023 Oct 18;23:994. doi: 10.1186/s12885-023-11492-z (PMC10585853; doi:10.1186/s12885-023-11492-z)
Supplement: Supplementary file 1 — Additional file 1. Supplementary Material. Supplementary material with the additional figures and tables. [file 12885_2023_11492_MOESM1_ESM.pdf]

Supplementary materials

Completeness of regional cancer registry data in Northwest Russia 2008-2017

Anton Barchuk, Rustam Tursun-zade, Ekaterina Nazarova, Yuri Komarov, Ekaterina Tyurina, Yulia Tumanova, Alexey Belyaev and Ariana Znaor

Supplementary table S1. Age-specific incidence rates per 100,000 for childhood cancer by sex and region, 2008–2012 and 2013-2017.

Supplementary table S2. Data sources and completeness estimates in eight regions in Northwest Russia (excluding St. Peterburg and Leningrad oblast), 2008–2017, by cancer site.

Supplementary table S3. Data sources and completeness estimates in St. Peterburg and Leningrad oblast, 2008–2017, by cancer site.

Supplementary table S4. Data sources and completeness estimates in eight regions in Leningrad oblast, 2008–2017, by cancer site.

Supplementary table S5. Cancer cases from the registry database and the national annual in regions of Northwest Russia, 2008–2017, by cancer site (excluding St. Petersburg and Leningrad oblast).

Supplementary table S6. Cancer cases from the registry database and the national annual report in St. Petersburg, 2008–2017, by cancer site.

Supplementary table S7. Cancer cases from the registry database and the national annual report in Leningrad oblast, 2008–2017, by cancer site.

Supplementary figure S1. The number of cases available from cancer registry databases in regions of Northwest Russia.

Supplementary figure S2. Age-standardized incidence rates per 100,000 for haematological malignancies (C81-C96), regions of the North-western Federal District (world population Segi-Doll, 1960).

Supplementary figure S3. Annual trends in age-standardized (world population Segi-Doll, 1960) incidence rates (log of rates) for selected sites in regions of Northwest Russia, 2000–2017.

Supplementary figure S4. Age-specific curves for cancers in regions of Northwest Russia (log of rates per 100,000), 2008–2017, compared to those in selected national and regional registries in Europe (Bulgaria, Czech Republic, Poland, Latvia, Lithuania, Estonia).

Supplementary table S1. Age-specific incidence rates per 100,000 for childhood cancer by sex and region, 2008–2012 and 2013-2017.

Region	Age group	Girls, rates per 100,000			Boys, rates per 100,000		
		2008-2012	2013-2017	Reference*	2008-2012	2013-2017	Reference*
Arkhangelsk oblast	0-4	17.2	17.9	>11.3 to <24.8	23.4	26.3	>13.1 to <27.1
Kaliningrad oblast		20.6	14.9		14.8	16.7	
Republic of Karelia		2.0	16.1		13.2	22.3	
Republic of Komi		13.4	18.2		25.7	29.3	
Leningrad oblast		26.5	13.6		15.8	12.9	
Murmansk oblast		21.7	21.3		26.4	21.9	
Novgorod oblast		16.3	19.5		25.6	24.7	
St. Petersburg		21.1	21.7		20.0	20.0	
Pskov oblast		20.0	20.0		15.3	28.5	
Vologda oblast		15.9	29.7		13.0	17.9	
Arkhangelsk oblast	5-9	7.1	10.7	>7.0 to <13.4	8.0	11.2	>9.1 to <15.9
Kaliningrad oblast		6.4	15.1		13.3	15.0	
Republic of Karelia		12.8	10.5		9.7	11.1	
Republic of Komi		6.6	12.3		7.9	13.3	
Leningrad oblast		5.6	6.4		5.9	3.3	
Murmansk oblast		7.1	7.5		4.9	9.7	
Novgorod oblast		11.5	3.8		20.1	18.7	
St. Petersburg		7.5	14.2		12.7	15.1	
Pskov oblast		9.8	3.8		14.4	7.2	
Vologda oblast		12.2	12.5		8.4	17.7	
Arkhangelsk oblast	10-14	6.4	11.3	>8.0 to <15.5	9.5	12.7	>9.2 to <16.9
Kaliningrad oblast		6.8	10.6		11.1	11.1	
Republic of Karelia		11.2	10.3		11.9	11.0	
Republic of Komi		9.7	12.8		9.3	13.1	
Leningrad oblast		12.6	2.1		8.0	6.1	
Murmansk oblast		8.9	4.2		9.4	15.8	
Novgorod oblast		19.9	18.6		27.2	21.5	
St. Petersburg		10.4	14.5		14.1	17.9	
Pskov oblast		19.1	18.3		8.3	7.9	
Vologda oblast		3.8	13.6		8.5	16.9	

* - Upper and lower deciles for childhood cancer incidence rates published in volume XI of CI5.

Supplementary table S2. Data sources and completeness estimates in eight regions in Northwest Russia (excluding St. Peterburg and Leningrad oblast), 2008–2017, by cancer site.

Site		Cases	Deaths*			Data sources (%)				Completeness (%)	
			Period	Cohort	M:I ratio†	C/P	C/P and D‡	DCO	DCI	Lincoln-Petersen estimator	Ajiki formula
Lip, oral, pharynx	C00-14	8,392	4,639	4,999	0.55	40.2	56.9	1.5	2.9	98.3	97.6
Oesophagus	C15	5,704	4,951	4,744	0.87	16.5	74.7	5.0	8.8	98.8	98.5
Stomach	C16	23,442	18,741	17,154	0.80	26.4	64.1	6.5	9.5	97.2	97.4
Colon, rectum, anus	C18-21	34,067	20,213	18,871	0.59	44.2	48.4	4.8	7.4	93.9	94.5
Liver	C22	2,967	4,091	2,604	1.38	11.3	58.3	15.3	30.4	96.2	112.0
Pancreas	C25	8,316	8,005	7,351	0.96	10.9	71.4	12.1	17.7	98.3	99.2
Larynx	C32	3,592	2,294	1,963	0.64	45.1	50.6	2.5	4.3	96.5	97.5
Trachea and lung	C33-34	30,216	25,719	23,457	0.85	21.9	66.7	7.1	11.4	97.5	97.7
Bone and cartilages	C40-41	850	683	472	0.80	44.2	48.9	4.6	6.8	94.4	98.2
Melanoma of skin	C43	5,471	1,853	1,804	0.34	67.0	31.7	0.6	1.3	95.9	97.4
Skin	C44	30,109	743	2,001	0.02	93.3	6.4	0.1	0.3	75.7	88.4
Soft tissues	C45-49	2,813	1,440	1,430	0.51	48.9	45.3	2.8	5.8	93.9	94.1
Breast	C50	30,284	10,315	7,756	0.34	74.2	24.2	0.8	1.5	91.5	97.0
Cervix uteri	C53	9,424	2,949	2,826	0.31	69.9	28.5	0.9	1.6	93.8	96.5
Corpus uteri	C54-55	10,751	2,906	2,684	0.27	74.9	22.2	1.8	2.8	83.2	92.1
Ovary	C56	7,018	3,849	3,588	0.55	48.6	46.9	2.5	4.5	95.5	96.2
Prostate	C61	13,973	4,805	4,648	0.34	66.0	30.4	2.3	3.6	89.2	92.9
Kidney	C64	11,844	4,411	4,185	0.37	63.7	30.8	2.4	5.4	85.6	90.3
Bladder	C67	7,676	3,242	2,992	0.42	60.6	35.0	2.7	4.4	90.6	93.7
Brain and CNS	C70-72	4,353	3,624	2,949	0.83	31.7	51.2	9.7	17.1	91.8	95.9
Hodgkins lymphoma	C81	1,688	518	385	0.31	76.7	19.4	2.1	3.9	73.4	90.8
Nonhodgkins lymphoma	C82-85	3,947	1,865	1,748	0.47	55.5	39.4	2.5	5.0	92.2	94.1
Leukaemia	C91-95	5,720	3,522	2,713	0.62	51.9	37.2	5.0	10.9	84.9	92.3
Other	a)	8,057	5,976	4,485	0.74	43.9	47.0	5.3	9.1	92.4	96.5
Other and ill-defined	b)	12,400	7,545	5,616	0.61	54.2	36.8	5.6	9.0	86.2	93.6
All sites	C00-C96	283,074	148,900	133,425	0.53	52.5	41.1	3.9	6.4	91.6	93.9
All sites but non-melanoma skin	C00-C96 (without C44)	252,965	148,158	131,424	0.59	47.6	45.3	4.3	7.1	92.9	94.6

a - 'Other' group includes following ICD-10 codes: C17, C23-24, C26, C30-31, C37-39,C51-52, C57-58, C60, C62-63, C65-66, C68, C90; b - 'Other and ill-defined' group includes following ICD-10 codes: C69, C73-80, C88, C96.

C/P - clinical/pathological notification only; C/P and D - clinical/pathological notification and death certificate; DCO - cases registered based on death certificates only; DCI - cases initially registered based on information from the death certificate and further investigated.

* - period cancer deaths were obtained from the civil registry for 2008-2017; cohort cancer deaths were obtained from the cancer registry database for patients diagnosed in 2008-2017; †- Mortality to incidence ratio was based on the number of deaths from the civil registry; ‡- all DCI cases were excluded, including those with clinical/pathological information.

Supplementary table S3. Data sources and completeness estimates in St. Petersburg, 2008–2017, by cancer site.

Site		Cases	Deaths*			Data sources (%)				Completeness (%)	
			Period	Cohort	M:I ratio†	C/P	C/P and D‡	DCO	DCI	Lincoln-Petersen estimator	Ajiki formula
Lip, oral, pharynx	C00-14	6,097	3,644	3,806	0.60	36.8	50.7	11.6	12.5	92.6	90.4
Oesophagus	C15	3,221	2,767	2,688	0.86	15.3	60.1	23.3	24.6	95.8	94.6
Stomach	C16	17,175	13,571	12,933	0.79	23.1	51.4	24.0	25.5	91.8	90.9
Colon, rectum, anus	C18-21	34,128	20,016	19,388	0.59	41.8	38.7	18.4	19.5	82.1	82.9
Liver	C22	3,523	3,788	3,127	1.08	8.8	40.6	48.1	50.6	92.3	107.2
Pancreas	C25	9,271	8,720	8,334	0.94	8.8	56.2	32.8	35.0	96.4	96.6
Larynx	C32	2,295	1,312	1,176	0.57	47.1	36.6	15.5	16.3	81.1	85.4
Trachea and lung	C33-34	21,483	17,868	16,798	0.83	20.3	51.2	27.0	28.5	92.0	91.9
Bone and cartilages	C40-41	640	475	376	0.74	40.6	38.4	19.7	20.9	81.6	90.8
Melanoma of skin	C43	5,384	2,036	1,936	0.38	63.8	28.7	6.9	7.6	79.4	86.5
Skin	C44	18,217	658	1,491	0.04	91.5	6.9	1.4	1.6	42.2	56.5
Soft tissues	C45-49	2,332	1,453	1,441	0.62	37.1	39.8	21.4	23.1	82.7	81.9
Breast	C50	28,609	11,568	8,938	0.40	68.0	22.7	8.9	9.2	66.4	85.0
Cervix uteri	C53	5,225	2,526	2,542	0.48	50.6	37.2	11.4	12.3	83.8	85.1
Corpus uteri	C54-55	9,493	3,312	3,279	0.35	64.6	23.7	10.8	11.8	64.8	75.1
Ovary	C56	6,452	3,874	3,863	0.60	39.4	42.1	17.3	18.5	85.6	84.9
Prostate	C61	13,624	4,814	4,811	0.35	63.6	25.4	10.4	11.0	69.1	77.4
Kidney	C64	9,158	3,858	3,696	0.42	57.0	25.8	16.3	17.2	64.4	71.5
Bladder	C67	6,518	2,657	2,701	0.41	57.1	28.3	13.5	14.7	70.6	75.0
Brain and CNS	C70-72	4,467	3,541	3,185	0.79	27.5	43.9	27.0	28.5	86.7	89.6
Hodgkins lymphoma	C81	1,390	401	306	0.29	77.1	14.9	7.5	8.0	47.7	78.6
Nonhodgkins lymphoma	C82-85	4,804	2,283	2,142	0.48	54.5	28.5	16.1	17.1	69.4	77.3
Leukaemia	C91-95	6,274	3,223	2,710	0.51	54.6	26.1	7.8	19.3	64.0	77.4
Other	a)	8,164	5,759	5,009	0.71	37.4	36.7	21.3	25.9	79.0	85.4
Other and ill-defined	b)	9,866	5,578	3,234	0.57	66.4	18.4	14.6	15.3	48.5	86.1
All sites	C00-C96	237,810	129,702	119,910	0.55	48.4	33.9	16.3	17.7	77.1	82.1
All sites but non-melanoma skin	C00-C96 (without C44)	219,593	129,045	118,419	0.59	44.8	36.1	17.6	19.1	79.4	83.5

a - ’Other’ group includes following ICD-10 codes: C17, C23-24, C26, C30-31, C37-39,C51-52, C57-58, C60, C62-63, C65-66, C68, C90; b - ’Other and ill-defined’ group includes following ICD-10 codes: C69, C73-80, C88, C96.

C/P - clinical/pathological notification only; C/P and D - clinical/pathological notification and death certificate; DCO - cases registered based on death certificates only; DCI - cases initially registered based on information from the death certificate and further investigated.

* - period cancer deaths were obtained from the civil registry for 2008-2017; cohort cancer deaths were obtained from the cancer registry database for patients diagnosed in 2008-2017; †- Mortality to incidence ratio was based on the number of deaths from the civil registry; ‡- all DCI cases were excluded, including those with clinical/pathological information.

Supplementary table S4. Data sources and completeness estimates in Leningrad oblast, 2008–2017, by cancer site.

Site		Cases	Deaths*			Data sources (%)				Completeness (%)	
			Period	Cohort	M:I ratio†	C/P	C/P and D‡	DCO	DCI	Lincoln-Petersen estimator	Ajiki formula
Lip, oral, pharynx	C00-14	1,650	1,317	653	0.80	60.4	33.8	3.8	5.8	87.6	98.4
Oesophagus	C15	872	1,222	565	1.40	34.6	49.5	9.4	15.8	91.2	105.4
Stomach	C16	3,695	4,691	2,112	1.27	42.5	44.1	9.0	13.4	88.7	103.3
Colon, rectum, anus	C18-21	6,106	5,397	2,364	0.88	61.2	28.2	7.1	10.6	74.4	98.4
Liver	C22	449	1,330	355	2.96	20.5	36.1	33.2	43.4	82.4	150.9
Pancreas	C25	1,279	2,468	957	1.93	24.9	52.1	16.3	23.1	92.0	114.4
Larynx	C32	677	573	215	0.85	68.1	25.7	3.5	6.2	77.8	98.8
Trachea and lung	C33-34	4,000	7,019	2,708	1.75	32.0	47.3	14.1	20.8	89.2	111.3
Bone and cartilages	C40-41	161	158	56	0.98	65.2	23.6	7.5	11.2	65.4	99.8
Melanoma of skin	C43	1,098	445	245	0.41	77.7	18.9	2.0	3.5	74.3	94.7
Skin	C44	4,440	247	189	0.06	95.5	3.6	0.4	0.9	26.2	85.3
Soft tissues	C45-49	455	361	141	0.79	68.8	25.3	4.2	5.9	77.8	98.4
Breast	C50	6,721	2,794	1,058	0.42	84.2	12.6	1.8	3.3	56.6	95.3
Cervix uteri	C53	1,633	922	426	0.56	73.9	23.1	1.8	3.1	83.4	97.6
Corpus uteri	C54-55	2,208	1,029	375	0.47	82.9	12.1	3.1	5.0	46.1	94.0
Ovary	C56	1,410	1,001	395	0.71	71.7	22.6	3.8	5.7	73.6	97.5
Prostate	C61	2,474	1,486	583	0.60	76.3	16.7	3.8	7.0	55.9	95.0
Kidney	C64	1,831	1,227	486	0.67	73.2	19.4	5.2	7.4	62.5	96.1
Bladder	C67	1,588	872	373	0.55	76.4	19.1	3.1	4.5	70.4	96.2
Brain and CNS	C70-72	543	952	309	1.75	42.9	31.3	19.9	25.8	71.4	114.9
Hodgkins lymphoma	C81	243	99	30	0.41	87.2	9.9	1.6	2.9	47.1	95.7
Nonhodgkins lymphoma	C82-85	635	621	198	0.98	68.3	20.5	7.9	11.2	58.3	99.7
Leukaemia	C91-95	582	845	255	1.45	55.3	26.1	12.7	18.6	64.3	107.1
Other	a)	1,451	1,464	553	1.01	61.3	25.3	8.3	13.4	66.3	100.1
Other and ill-defined	b)	2,334	1,529	522	0.66	77.6	17.3	3.3	5.1	63.2	97.1
All sites	C00-C96	48,535	40,072	16,123	0.83	66.6	24.5	5.9	8.9	70.4	97.9
All sites but non-melanoma skin	C00-C96 (without C44)	44,095	39,825	15,934	0.90	63.7	26.6	6.5	9.8	72.9	98.8

a - 'Other' group includes following ICD-10 codes: C17, C23-24, C26, C30-31, C37-39,C51-52, C57-58, C60, C62-63, C65-66, C68, C90; b - 'Other and ill-defined' group includes following ICD-10 codes: C69, C73-80, C88, C96.

C/P - clinical/pathological notification only; C/P and D - clinical/pathological notification and death certificate; DCO - cases registered based on death certificates only; DCI - cases initially registered based on information from the death certificate and further investigated.

* - period cancer deaths were obtained from the civil registry for 2008-2017; cohort cancer deaths were obtained from the cancer registry database for patients diagnosed in 2008-2017; †- Mortality to incidence ratio was based on the number of deaths from the civil registry; ‡- all DCI cases were excluded, including those with clinical/pathological information.

Supplementary table S5. Cancer cases from the registry database and the national annual report in Northwest Russia, 2008–2017, by cancer site (excluding St. Petersburg and Leningrad oblast).

		Number of cases		Difference	
Site	ICD-10	Registry	National report	Absolute	Relative (%)
Lip, oral, pharynx	C00-14	8392	7890	502	6.0
Oesophagus	C15	5704	5601	103	1.8
Stomach	C16	23442	22912	530	2.3
Colon, rectum, anus	C18-21	34067	33076	991	2.9
Liver	C22	2967	3506	-539	-18.2
Pancreas	C25	8316	8163	153	1.8
Larynx	C32	3592	3501	91	2.5
Trachea and lung	C33-34	30216	29475	741	2.5
Bone and cartilages	C40-41	850	780	70	8.2
Melanoma of skin	C43	5471	5130	341	6.2
Skin	C44	30109	29632	477	1.6
Soft tissues	C45-49	2813	1792	1021	36.3
Breast	C50	30284	29652	632	2.1
Cervix uteri	C53	9424	9199	225	2.4
Ovary	C56	7018	7012	6	0.1
Prostate	C61	13973	13575	398	2.8
Kidney	C64	11844	11324	520	4.4
Bladder	C67	7676	7401	275	3.6
Brain and CNS	C70-72	4353	4065	288	6.6
Haematological malignancies	C81-C95	11355	12846	-1491	-13.1
All sites	All sites	283074	276058	7016	2.5

Supplementary table S6. Cancer cases from the registry database and the national annual report in St. Petersburg, 2008–2017, by cancer site.

		Number of cases		Difference	
Site	ICD-10	Registry	National report	Absolute	Relative (%)
Lip, oral, pharynx	C00-14	6097	5303	794	13.0
Oesophagus	C15	3221	2956	265	8.2
Stomach	C16	17175	15524	1651	9.6
Colon, rectum, anus	C18-21	34128	30974	3154	9.2
Liver	C22	3523	3255	268	7.6
Pancreas	C25	9271	8262	1009	10.9
Larynx	C32	2295	2061	234	10.2
Trachea and lung	C33-34	21483	19149	2334	10.9
Bone and cartilages	C40-41	640	556	84	13.1
Melanoma of skin	C43	5384	4838	546	10.1
Skin	C44	18217	17148	1069	5.9
Soft tissues	C45-49	2332	1136	1196	51.3
Breast	C50	28609	25640	2969	10.4
Cervix uteri	C53	5225	4722	503	9.6
Ovary	C56	6452	6124	328	5.1
Prostate	C61	13624	11753	1871	13.7
Kidney	C64	9158	8216	942	10.3
Bladder	C67	6518	5846	672	10.3
Brain and CNS	C70-72	4467	3988	479	10.7
Haematological malignancies	C81-C95	12468	12814	-346	-2.8
All sites	All sites	237810	214506	23304	9.8

Supplementary table S7. Cancer cases from the registry database and the national annual report in Leningrad oblast, 2008–2017, by cancer site.

		Number of cases		Difference	
Site	ICD-10	Registry	National report	Absolute	Relative (%)
Lip, oral, pharynx	C00-14	1650	1883	-233	-14.1
Oesophagus	C15	872	1106	-234	-26.8
Stomach	C16	3695	4728	-1033	-28.0
Colon, rectum, anus	C18-21	6106	7561	-1455	-23.8
Liver	C22	449	766	-317	-70.6
Pancreas	C25	1279	1870	-591	-46.2
Larynx	C32	677	746	-69	-10.2
Trachea and lung	C33-34	4000	6186	-2186	-54.6
Bone and cartilages	C40-41	161	219	-58	-36.0
Melanoma of skin	C43	1098	1114	-16	-1.5
Skin	C44	4440	5379	-939	-21.1
Soft tissues	C45-49	455	260	195	42.9
Breast	C50	6721	6705	16	0.2
Cervix uteri	C53	1633	1704	-71	-4.3
Ovary	C56	1410	1459	-49	-3.5
Prostate	C61	2474	3065	-591	-23.9
Kidney	C64	1831	2075	-244	-13.3
Bladder	C67	1588	1521	67	4.2
Brain and CNS	C70-72	543	733	-190	-35.0
Haematological malignancies	C81-C95	1460	1984	-524	-35.9
All sites	All sites	48535	57556	-9021	-18.6

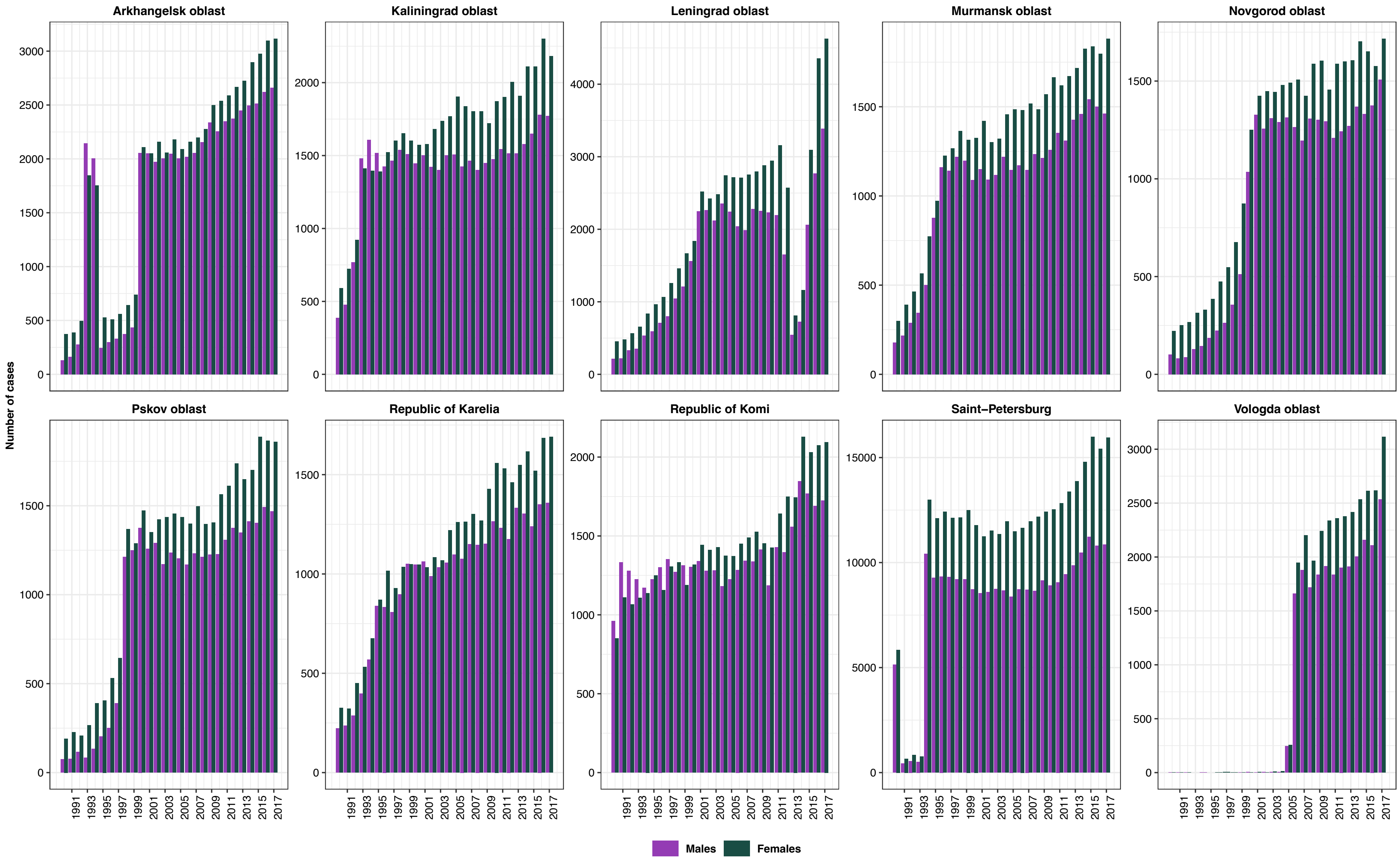

Supplementary figure S1. The number of cases available from cancer registry databases in regions of Northwest Russia.

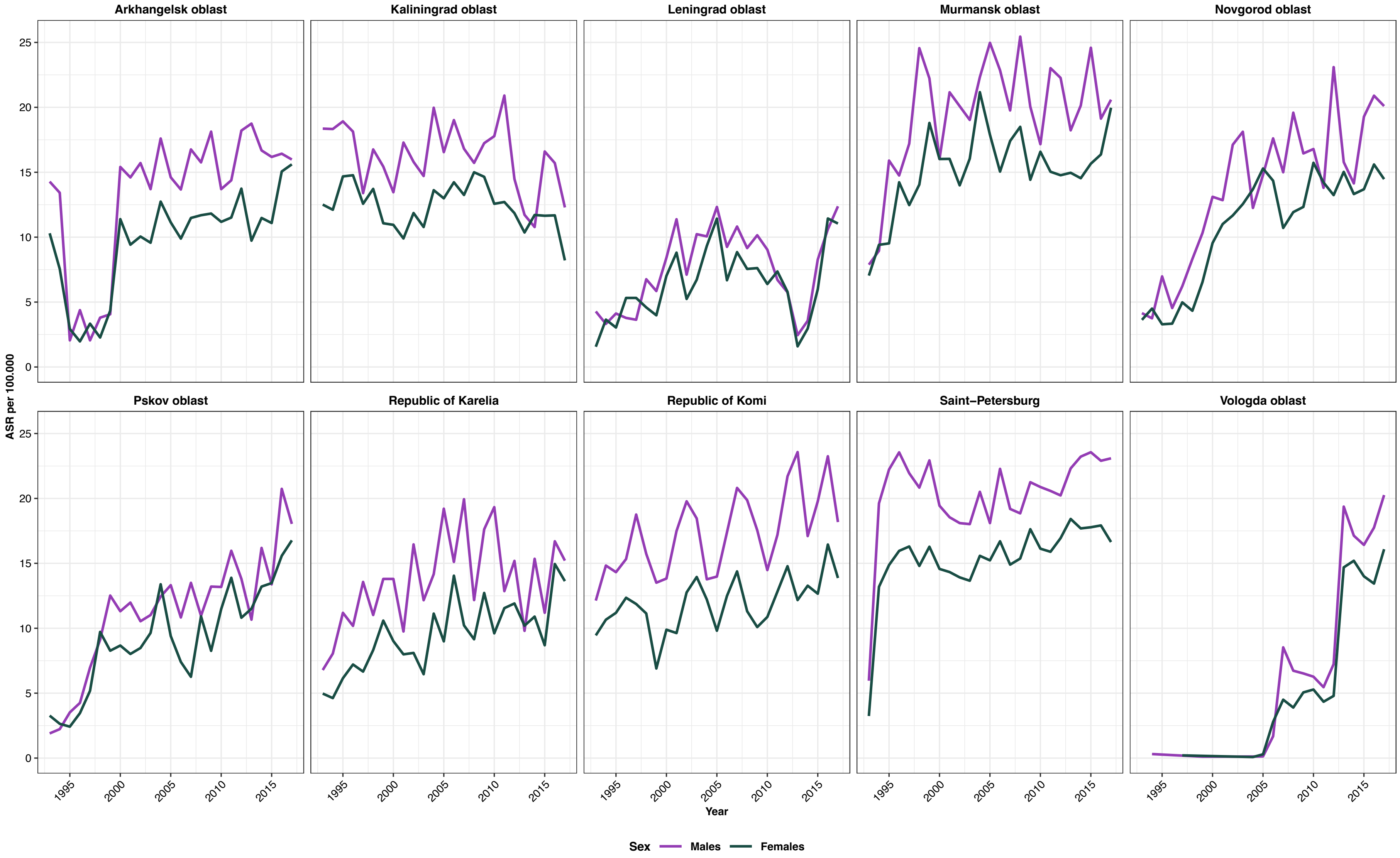

Supplementary figure S2. Age-standardised incidence rates per 100,000 for haematological malignancies (C81-C96), regions of Northwest Russia (world population Segi-Doll, 1960).

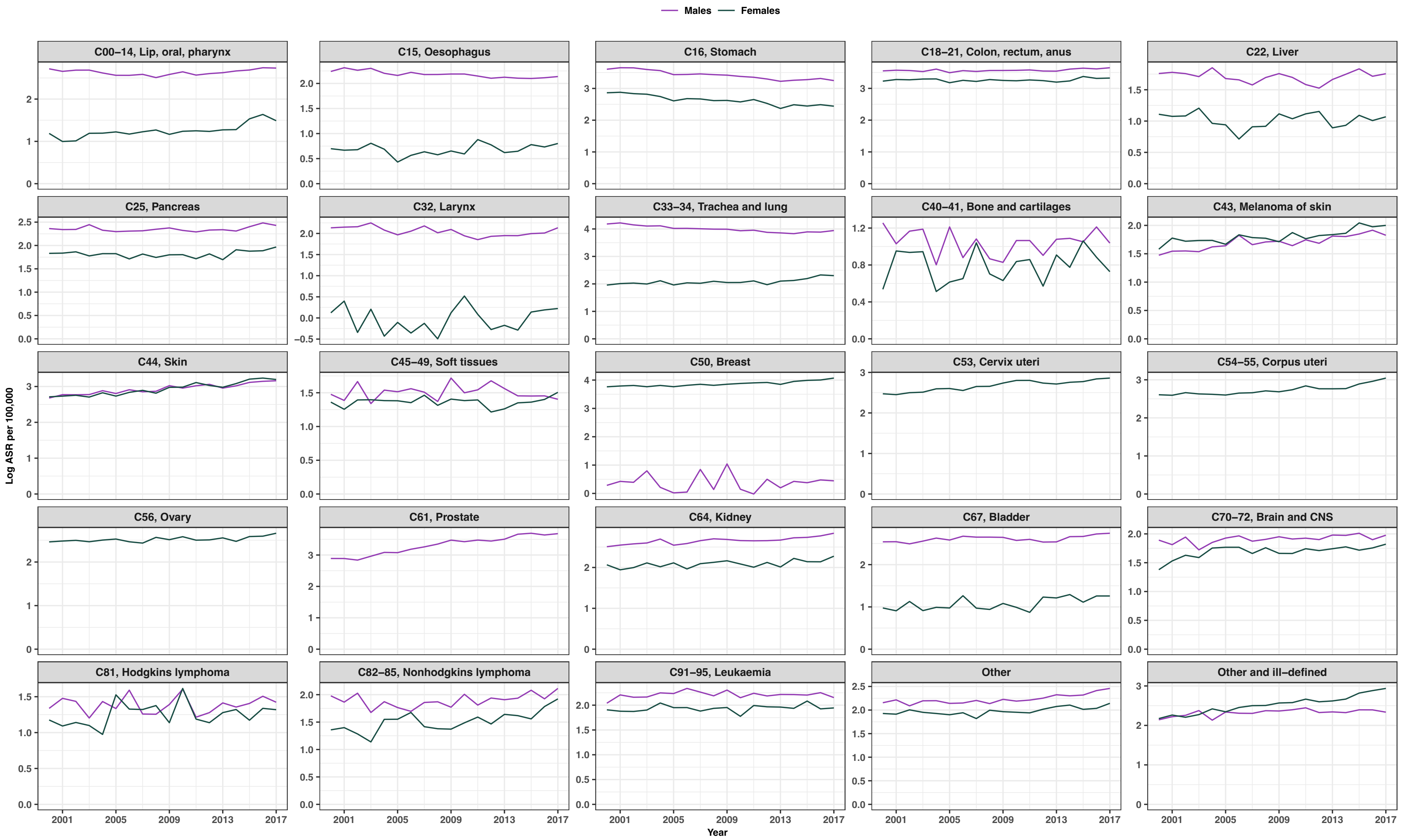

Supplementary figure S3. Annual trends in age-standardized (world population Segi-Doll, 1960) incidence rates (log of rates) for selected cancer types, in regions of Northwest Russia, 2000–2017).

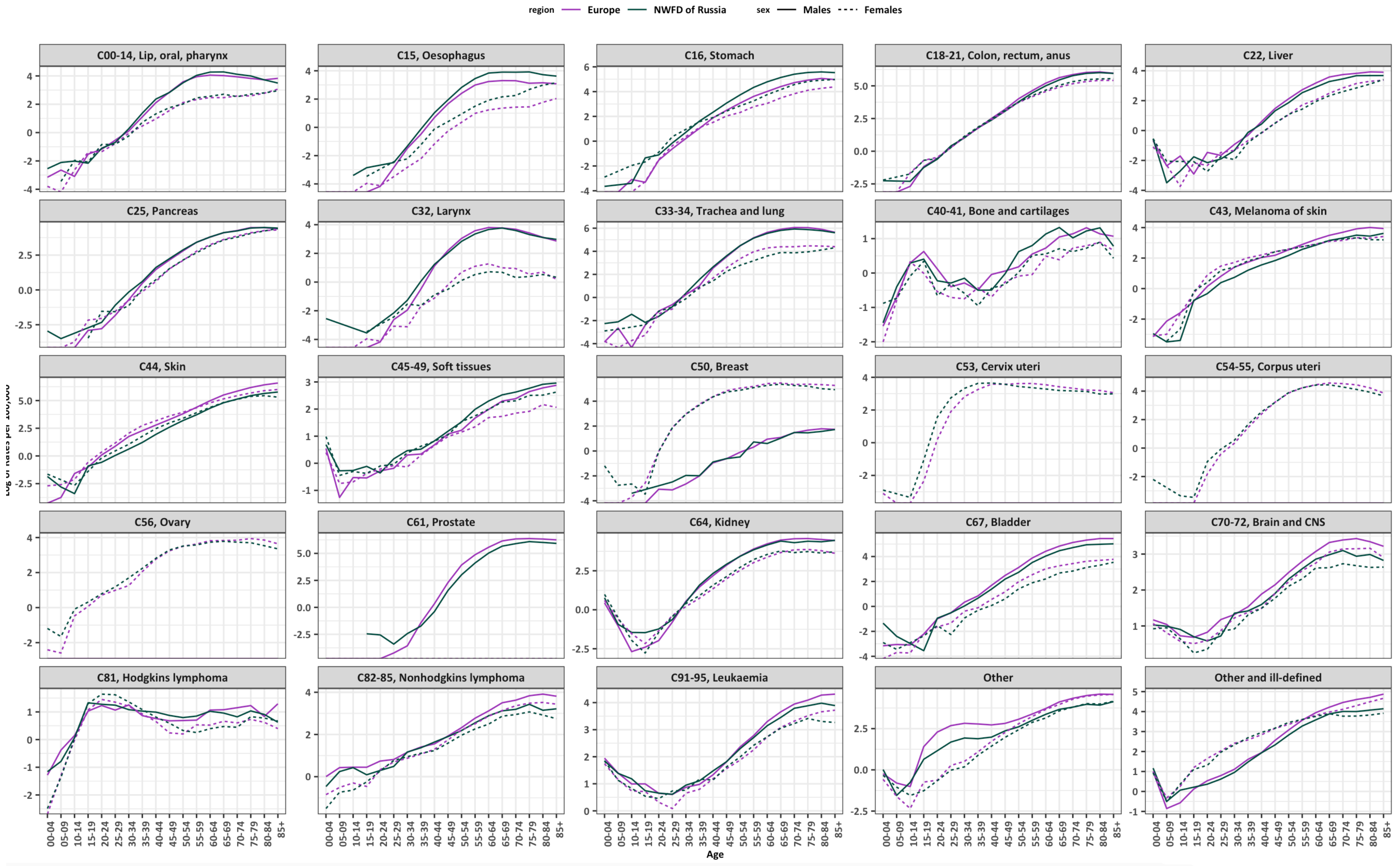

Supplementary figure S4. Age-specific curves for cancers in all ten regions of Northwest Russia (log of rates per 100,000), 2008–2017, compared to those in selected national and regional registries in Eastern Europe (Bulgaria, Czech Republic, Poland, Latvia, Lithuania, Estonia). Linetype represents gender (solid - men, dashed - women), and colors represent different regions (purple - regional registries in Eastern Europe and green - ten regions of Northwest Russia).
